# Controlled prophylactic normothermia

**DOI:** 10.1186/cc11268

**Published:** 2012-06-07

**Authors:** Gregor Broessner, Marlene Fischer, Bettina Pfausler, Erich Schmutzhard

**Affiliations:** 1 Neurologic Intensive Care Unit, Medical University Innsbruck, Austria

## Introduction

Fever is a very frequent complication of intensive care treatment and an independent predictor of unfavourable outcome and mortality in most patients with an acute severe neurologic injury. Today not only treatment but even more prevention of fever has become the focus of intensivists.

Preliminary animal data for the beneficial neuroprotective effect of therapeutic hypothermia could not satisfyingly be reproduced in patients raising questions about the possible side effects of hypothermia. Controlled prophylactic normothermia (36.5°C) prevents secondary injury through consequent treatment of fever and limits dose-dependent side effects through therapeutic hypothermia. Novel endovascular and gel-pad surface cooling measures have shown to be feasible and efficacious in inducing and maintaining even long-term controlled normothermia.

## Rationale for treatment of fever

Fever is one of the most frequent complications of intensive care treatment. Up to 90% of patients develop at least one febrile episode within 7 days after being admitted to an ICU [1]. First of all, fever has always to be interpreted as a sign of an infection. Thus temperature modulation by any means has to include a strict protocol identifying any source of infection followed by a straightforward treatment approach. There is widespread consensus that fever alone is associated with unfavourable outcome. This consensus is a result of animal and human data over the past decades. In a meta-analysis conducted by Greer and colleagues including more than 14,000 patients, fever alone was a significant and independent predictor of morbidity and mortality across such different diseases entities as ischaemic stroke, haemorrhagic stroke and traumatic brain injury [2]. Increasing evidence from animal and human studies suggests that fever, irrespective of its cause, can directly and adversely affect neurological outcome in various types of neurological injury [3].

The pathophysiological mechanisms by which fever affects patient outcome are discussed, controversially comprising increase of metabolic demand (under circumstances of reduced supply), production of free radicals, local thermopooling, disruption of the blood-brain barrier, intracranial pressure (ICP) elevation, increased enzymatic inhibition of protein kinases, and worsened cytoskeletal proteolysis [1,3].

## Concept of controlled prophylactic normothermia

The aggressive treatment of fever in any patient with a severe acute neurologic injury has become increasingly important and is now the focus of many prospective studies including such patients. Whether the reduction of hyperthermia alone or even controlled hypothermia should be the treatment goal is still under debate and there are pros and cons for either approach. The enthusiastic preliminary results from animal hypothermia studies could not be satisfyingly reproduced when implemented in human controlled trials, shifting the focus on the possible side effects of hypothermia. Today there is a whole body of evidence that the potentially neuroprotective effects of hypothermia can be significantly diminished by its inherent side effects. It could be shown that hypothermia may lead to increased rate of infections, hypotension, shivering, disturbances in blood clotting, rewarming injuries and significant changes in pharmacokinetics and pharmacodynamics possibly limiting outcome effects of the treated patients [4-8]. Aggressive treatment of fever in the ICU without risk elevation through the side effects of therapeutic hypothermia led to the concept of controlled prophylactic normothermia. This concept is based upon strict control of body core temperature with a target of 36.5°C beginning as early as possible with the goal of complete fever prevention. Prophylactic controlled normothermia can therefore not be compared with the normothermia control group of most randomised trials since this novel approach aims to control temperature prophylactically and is therefore not only treatment of fever.

## Experiences from clinical trials

Controlled prophylactic normothermia cannot be achieved through conventional temperature control measures including NSAIDS and conventional cooling blankets [5,9]. In a controlled trial conducted by our study group, reduction of fever burden (that is, body core temperature >37.9°C) was significantly higher in the endovascular cooling group than in the control group although strictly following a predefined fever management protocol including NSAIDS, opioids and conventional cooling blankets in patients with severe cerebrovascular diseases [5]. In this trial an endovascular cooling catheter was placed in the subclavian vein and prophylactic normothermia was maintained over 168 hours in patients with ischaemic stroke and intracerebral haemorrhage and over 336 hours in patients with spontaneous subarachnoid haemorrhage[5]. Safety evaluation revealed no relevant increase in direct device-related adverse events in the endovascular group.

Although there was significant decrease of the fever burden in the device group no difference could be found in the long-term, 6-month, patient follow-up. This lack of outcome efficacy may be attributed due to the increased rate of infectious complications in the device group again pointing out that state-of-the-art temperature modulation has to be combined with a standardised surveillance of infections [6].

In patients with severe traumatic brain injury (GCS ≤8) direct measurement of brain temperature together with ICP is possible [10]. Since fever may deteriorate elevated ICP, prophylactic controlled normothermia should be evaluated in this patient population. In a small pilot trial, brain temperature under endovascular cooling showed that even under normothermia (36.5°C) brain temperature almost reaches body core temperature [11]. This is of utmost interest as brain temperature exceeds body core temperature even under physiological circumstances and even more under fever with a peak gap >2°C [10]. As the brain is the target tissue of all neuroprotectants, it is now evident that even controlled endovascular normothermia can significantly lower brain temperature in TBI patients.

Others could achieve normothermia through a novel surface cooling device using gel-coated energy transfer pads, directly applied to the skin in patients with spontaneous subarachnoid haemorrhage and severe traumatic brain injury [9,12].

## Questions to be addressed in future trials

What is the optimal target temperature and duration for temperature modulation in various disease entities? What is the appropriate approach to inherent complications such as shivering, infections, pharmacodynamic and pharmacokinetic disturbances? Are surface and endovascular treatment approaches equivalent?

## Conclusion

Controlled prophylactic normothermia is not only fever reduction but aims to strictly maintain the body temperature at 36.5°C. Induced as early as possible the duration should cover the acute phase of the neurological injury, minimising secondary additional neurological impairment though prevention of fever. Endovascular and surface cooling measures using gel-coated pads have shown to be efficacious and feasible in inducing and maintaining normothermia whereas conventional temperature control measures including NSAIDs and conventional cooling blankets are not sufficient to prevent fever. Side effects known from therapeutic hypothermia such as shivering and increased rate of infections also may occur under controlled prophylactic normothermia, although to a lesser extent. Nevertheless, a consequent prevention of those limiting factors should be kept in mind when applying controlled prophylactic normothermia.
